# Science Avant-Garde

**DOI:** 10.3201/eid1811.AC1811

**Published:** 2012-11

**Authors:** Polyxeni Potter

**Affiliations:** Author affiliation: Centers for Disease Control and Prevention, Atlanta, Georgia, USA

**Keywords:** art science connection, emerging infectious disease, art and medicine, Science Avant-Garde, Paul Signac, La salle à manger, Breakfast, DNA sequences, epidemiologic techniques, pathogen databases, about the cover

**Figure:**
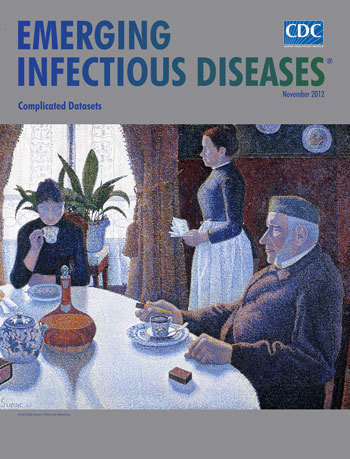
**Paul Signac (1863–1935) *La salle à manger*, *Breakfast* (1886–1887) Oil on canvas (89.5 cm × 116.5 cm)** Kröller-Müller Museum, Otterlo, the Netherlands www.kmm.nl/

“To think that the neoimpressionists are painters who cover canvases with little multicolored spots is a rather widespread mistake,” wrote Paul Signac in his manifesto of their movement. “This mediocre dot method has nothing to do with the aesthetic of the painters we are defending here, nor with the technique of divisionism they use.” Signac was referring to, among others, Georges Seurat, who in trying to systematize the optical discoveries of the impressionists, had taken a scientific approach to painting, one based on color theory. His goal, he once said, was to make “modern people, in their essential traits, move about as if on friezes, and place them on canvases organized by harmonies of color, by directions of the tones in harmony with the lines, and by the directions of the lines.”

Signac wholeheartedly adopted Seurat’s invention, pointillism or divisionism, though he considered it simply a means of expression, a way to apply paint to the canvas. “The dot is nothing more than a brushstroke, a technique. And like all techniques, it does not matter much.” The idea was to render the surface of a painting more vibrant, to maximize the brilliance of color. But the methodical scientific technique alone did not “guarantee luminosity or the intensity of colors or harmony. This is due to the fact that complementary colors are favorable to and intensify each other when they are blended, even optically. A red surface and a green surface, when adjacent, stimulate one another. Red dots blended into green dots produce a gray and colorless whole.”

“My family wanted me to become an architect, but I preferred drawing on the banks of the Seine rather than in a workshop of the École des Beaux-Arts,” wrote Signac. A visit to an exhibition of Claude Monet in 1880 was a life-changing experience. After a brief stint at the Collège Rollin, he set out to become a painter, which he did, a stellar one and self-taught. His earliest work was filled with energy. “It consisted in pasting reds, greens, blues, and yellows, without much care but with enthusiasm.” When he lost his father to tuberculosis, his financially stable and supportive family saw to it he did not have to make ends meet. The same year, at age 17, he bought a painting by Paul Cézanne. His own first painting was dated a year later.

Signac spent most of his life in and around Paris where he was born. He was interested in science, literature, and politics. He was a writer, whose poetic sensitivity found its way into landscape painting. He was an avid traveler. His *Olympia*, a boat named after Édouard Manet’s famed nude, took him to Italy, Holland, and Constantinople. He often stopped to paint Mediterranean ports and scenery, immortalizing the French coast in watercolors painted *en plein air*. Although he experimented with oils, pen and ink, etchings, and lithographs, watercolor, “a playful game,” was the mainstay of his life’s work.

“I have seen Signac, and it has done me quite a lot of good,” wrote Vincent van Gogh to his brother Theo. “He was so good and straightforward and simple …. I found Signac very quiet though he is said to be violent; he gave me the impression of someone who has balance and poise.” Signac’s irrepressible vitality and exuberance, his love of action and the outdoors, and a native combativeness were at times misunderstood, but not by his many friends, an array of artists and anarchists. Unassuming and ragged in his sailor’s garb, he was often at sea or at his home in St. Tropez, a meeting place for the exchange and promotion of artistic ideas. Signac equated social revolution with artistic freedom. “The anarchist painter is not the one who will create anarchist pictures, but he who, without desire for recompense, will fight with all his individuality against official bourgeois conventions by means of a personal contribution.” At age 21, Signac became, along with Georges Seurat and others, cofounder of the Société des Artistes Indépendants, a group intended to provide opportunities for exhibiting avant-garde works away from the rigid cultural politics of the Paris Salon. President of the society from 1908 until his death, Signac encouraged young artists by exhibiting controversial works. Meanwhile, with Seurat, he set off to articulate the theories of neoimpressionism. After the untimely death of Seurat from respiratory infection at age 31, Signac became the sole advocate and leader of the movement.

Signac took Seurat’s theories to a new level. Armed with watercolor sketches from nature, he moved the studio indoors and used mosaic-like squares of pure color to compose large scenes that would influence the works of van Gogh and Gauguin, inspire Matisse, and affect the evolution of future art movements, from fauvism to cubism. Signac was tireless in explaining divisionism. “In order to listen to a symphony, you don’t sit in the middle of the orchestra but in the position where the sounds from the various instruments mingle, creating the harmony desired by the composer. Similarly, faced by a ‘divided’ painting, it is best to first stand at a sufficient distance in order to absorb the whole, before moving closer to study the chromatic effects up close.” For the first 20 years of his career, he received little recognition and neoimpressionism received negative criticism, even by those who initially supported it. He died of septicemia in Paris at 72.

*La salle à manger*, *Breakfast,* on this month’s cover, is from a series of views inside contemporary interiors with figures usually posed in stiff profile. Signac valiantly sought art solutions in the scientific process, the precise observation of color tones in close proximity. And, moving away from the subjectivity of impressionism and the passing moment, he searched the small particles of color for truth.

Always interested in human psychology and social justice, he looked for them in home interiors, as he curiously observed the urban middle-class. In *Breakfast*, he spied on them from outside the room, inviting the viewer to do the same. With scant interest in perspective, he placed the human figures on a grid, same as all other objects. Frozen in space and time, these figures were not persons but social types: the retired bourgeois, the maid, the housewife. Uninterested in each other, they played roles, their performance an indictment of society and marriage, which inhibits the development of individual personality.

“By exclusive use of … pure colors, by methodical division and by observing the scientific theory of colors, [the neoimpressionist] guarantees maximal luminosity, color, and harmony to an unprecedented degree,” Signac wrote. The painter’s vision applies neatly to today’s rapid advancement of genome technologies that, by providing tiny bits of data on disease-causing microbes, promise to improve the canvas of clinical and public health laboratory investigations and lower the costs.

The latest genome DNA sequencers generate detailed and robust information for clinical and public health laboratories and could spawn a global system of linked databases of pathogen genomes to ensure more efficient detection, prevention, and control of endemic and emerging diseases and all manner of outbreaks. Even as these new genomic tools enhance diagnosis, they decrease the use of culture and molecular methods that produce data currently critical for epidemiologic investigation. Careful application of current epidemiologic techniques teases apart the dynamic interaction of infectious diseases that drive total illness and death rates up or down, even in outbreaks with universal exposure. New genome-backed epidemiologic approaches will be needed as sequencers replace culture and molecular techniques so this ability is not lost.

“To ensure optical mixture, the neoimpressionists were forced to use brushwork of a small scale so that, when standing back sufficiently, different elements could reconstitute the desired tint and not be perceived in isolation.” In genomics approaches, likewise, field epidemiologists must use alternative data sources or original techniques to capture the unique characteristics that tie together the epidemiologically related whole. Without these, the bits provided by the precise genomic tools would only create “industrial art,” a canvas without valuable content, aesthetics achieved, in Signac’s words, by “empirical formulae and dishonest or silly advice.”
